# Sex differences in contributors to coronary microvascular dysfunction

**DOI:** 10.3389/fcvm.2023.1085914

**Published:** 2023-01-24

**Authors:** Alan C. Kwan, Janet Wei, David Ouyang, Joseph E. Ebinger, C. Noel Bairey Merz, Daniel Berman, Susan Cheng

**Affiliations:** ^1^Department of Cardiology, Smidt Heart Institute, Los Angeles, CA, United States; ^2^Barbara Streisand Women’s Heart Institute, Los Angeles, CA, United States; ^3^Department of Imaging, Cedars Sinai Medical Center, Los Angeles, CA, United States

**Keywords:** coronary microvascular dysfunction (CMD), cardiac MRI (CMR), myocardial perfusion reserve index, sex differences, cardiac fibrosis, cardiac remodeling

## Abstract

**Background:**

Coronary microvascular dysfunction (CMD) has differences in prevalence and presentation between women and men; however, we have limited understanding about underlying contributors to sex differences in CMD. Myocardial perfusion reserve index (MPRI), as semi-quantitative measure of myocardial perfusion derived from cardiac magnetic resonance (CMR) imaging has been validated as a measure of CMD. We sought to understand the sex differences in the relations between the MPRI and traditional measures of cardiovascular disease by CMR.

**Methods:**

A retrospective analysis of a single-center cohort of patients receiving clinical stress CMR from 2015 to 2022 was performed. Patients with calculated MPRI and no visible perfusion defects consistent with obstructive epicardial coronary disease were included. We compared associations between MPRI versus traditional cardiovascular risk factors and markers of cardiac structure/function in sex-stratified populations using univariable and multivariable regression models.

**Results:**

A total of 229 patients [193 female, 36 male, median age 57 (47–67) years] were included in the analysis. In the female population, no traditional cardiovascular risk factors were associated with MPRI, whereas in the male population, diabetes (β: −0.80, *p* = 0.03) and hyperlipidemia (β: −0.76, *p* = 0.006) were both associated with reduced MPRI in multivariable models. Multivariable models revealed significant associations between reduced MPRI and increased ascending aortic diameter (β: −0.42, *p* = 0.005) and T1 times (β: −0.0056, *p* = 0.03) in the male population, and increased T1 times (β: −0.0037, *p* = 0.006) and LVMI (β: −0.022, *p* = 0.0003) in the female population.

**Conclusion:**

The findings suggest different underlying pathophysiology of CMD in men versus women, with lower MPRI in male patients fitting a more “traditional” atherosclerotic profile.

## Introduction

Coronary microvascular dysfunction (CMD) encompasses a wide variety of pathology though which the microcirculation of the heart is compromised, causing ischemia and infarction in the absence of obstructive epicardial coronary disease (1). Prevalence estimates vary significantly based on demography and clinical subgrouping but are known to vary between men and women (2). Estimates of CMD prevalence ranges between 34–66% in women and 14–60% in men in large cohort studies of patients with ischemic symptoms and non-obstructive coronary angiograms (3–5). CMD is associated with significantly increased risk of major adverse cardiovascular events (MACE) in both men and women, though the most frequent presentation of MACE in men is mortality and in women heart failure admission (5). CMD has recently been identified as a factor in the pathogenesis of heart failure with preserved ejection fraction (HFpEF) (6, 7).

Coronary microvascular dysfunction can be identified invasively, considered the gold standard, or non-invasively through stress cardiac MRI or positron emission tomography (PET) perfusion studies (8). The myocardial perfusion reserve index (MPRI) is a cardiac magnetic resonance (CMR) imaging-based semi-quantitative measurement of myocardial perfusion which has been extensively validated in the context of CMD (9–12). Understanding of the observed differences in CMD prevalence, associated outcomes and manifestations between men and women, suggests that investigation of sex-differences in CMD is necessary. In this study, we examine the relation of traditional cardiovascular risk factors and cardiac structure and function with MPRI in clinical patients with comprehensive stress cardiac MRI and no evidence of obstructive epicardial coronary artery disease (CAD) to identify correlates of CMD in both total and sex-stratified analyses.

## Materials and methods

### Study sample

The study sample consisted of a single-center cohort of CMR performed for clinical indications from 2015 to 2022. Clinical indications were diverse and included cardiomyopathies, arrhythmias, abnormal testing, and suspected coronary artery disease among other disease-specific indications. The sample was restricted to participants who received a comprehensive CMR protocol with contrast and stress agent (adenosine or regadenoson). In order to avoid confounding by epicardial CAD and to more clearly measure CMD, we included only those with measured MPRI and excluded participants who had visible regional perfusion abnormalities suggesting epicardial stenoses as the course (*N* excluded = 196). All study protocols were approved by the Cedars-Sinai Medical Center Institutional Review Board, and informed consent was waived for this retrospective study.

### Clinical assessment

**FIGURE 1:**
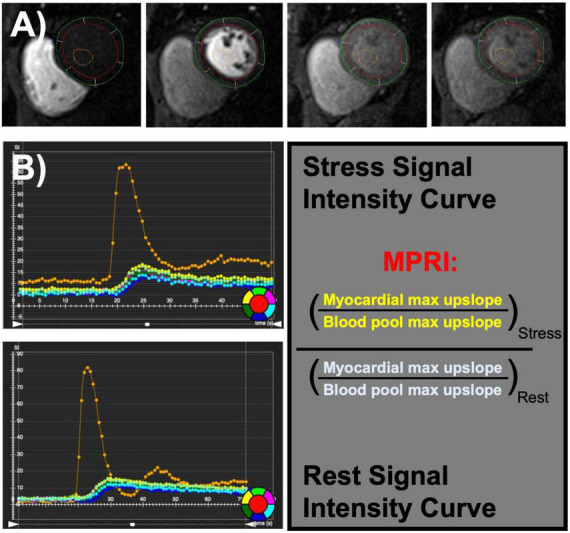
Coronary microvascular dysfunction can be assessed using semi-quantitative evaluation of first-pass perfusion images **(A)** during stress and rest cardiac magnetic resonance imaging (MRI). Myocardial perfusion reserve index (MPRI) is calculated by the maximal upslope of myocardial intensity normalized by maximal upslope of blood pool intensity at stress divided by rest **(B)**.

Studies were performed on an Avanto 1.5 T scanner. Standard clinical assessment was performed at the time of the examination including study stress characteristics (stress agent, max heart rate, and blood pressure), patient demographics, traditional cardiovascular risk factors [age, sex, diabetes, hypertension, hyperlipidemia, family history of premature coronary artery disease (CAD), and smoking], cardiovascular structure [diastolic volumes, atrial size, aortic diameter, left ventricular mass index (LVMI), and relative wall thickness], function [left and right ventricular ejection fraction (LVEF, RVEF), and cardiac output], composition (T1 times, T2 times), and MPRI. Patient symptoms were also assessed by standardized survey. Perfusion images were performed using a standard gradient-echo echo-planar imaging hybrid sequence and were used to calculate the MPRI (11). The MPRI was calculated as part of standard clinical reading of first-pass perfusion by taking the ratio of the maximal signal upslope in the blood pool versus the left ventricular myocardial signal upslope in a mid-ventricular short axis at stress, divided by at rest (Figure 1) using commercially available software (Vitrea Medical Imaging Software, Canon Medical, Minnetonka, MN, USA). Previous validation of MPRI has shown it be a marker of coronary microvascular dysfunction in the absence of obstructive epicardial disease (9–12).

### Statistical analysis

Descriptive statistics were calculated with and without sex stratification, with differences between male and female populations estimated with Wilcoxon rank sum test and chi-squared testing. Linear regression with sex stratification was used to assess the relation of MPRI (primary outcome) with exposures groups including demographic/risk factors and structural characteristics. Univariable and multivariable testing was performed after assessment for multicollinearity by calculation of variance inflation factor (VIF) with elimination of factors with VIF >10, and final models determined by backward stepwise selection by Akaike information criterion. Secondary analyses were performed in the aggregate population to assess the effects of acquisition factors, and also using LVMI and MPRI/LVMI as the primary outcome to assess effects of normalization by structural features on predictors. Statistical analyses were performed using R version 4.2.1, with statistical significance defined as a two-tailed *p* < 0.05.

## Results

The overall population consisted of 229 patients [85% female, median age 57 (IQR 47, 67) years]. There was a high prevalence of cardiovascular risk factors, including diabetes and hypertension in approximately half of the patients (48 and 51%, respectively) and lower rates of smoking and family history of premature CAD. Cardiac structural features were largely within normal ranges, and the median MPRI was 2.50 (IQR 2.10, 3.15). In comparisons between male and female populations, hyperlipidemia was the only risk factor with a significantly higher prevalence in men. Most of the structure/function factors were different between the male and female populations including ejection fractions, end diastolic volumes, aortic diameter, cardiac output, relative wall thickness, and LVMI. Notably, T1 and T2 times were not different in this population, and MPRI was also not significantly different between the populations (Table 1). Symptom assessment revealed different presentations, with men reporting symptoms consistent with atypical angina 44% of the time, whereas women reported atypical angina 84% of the time. Typical angina was seen 6% of the time in men, and 3% of the time in women. Non-anginal chest pain was reported <1% of the time in women, and 6% of the time in men.

**TABLE 1:** Demographic, traditional risk factors, and cardiac imaging characteristics overall and by sex.

	Overall, *N* = 229	Females, *N* = 193	Males, *N* = 36	*P*-value (male vs. female)
Age (years)	57 (47, 67)	57 (47, 67)	60 (47, 68)	0.6
Hypertension	109 (48%)	87 (45%)	22 (61%)	0.077
Diabetes	16 (7.0%)	11 (5.7%)	5 (14%)	0.14
Hyperlipidemia	117 (51%)	93 (48%)	24 (67%)	**0**.**042**
Family history	58 (25%)	52 (27%)	6 (17%)	0.2
Smoking	7 (3.1%)	6 (3.1%)	1 (2.8%)	>0.9
**Structural imaging measures**				
LV mass index (g/m^2^)	42 (35, 50)	40 (35, 46)	52 (45, 63)	<0.001
Relative wall thickness	0.36 (0.30, 0.43)	0.35 (0.30, 0.42)	0.39 (0.34, 0.46)	0.013
LV EDV (cc)	114 (94, 139)	112 (92, 130)	142 (122, 170)	<0.001
RV EDV (cc)	115 (93, 128)	109 (91, 121)	142 (115, 167)	<0.001
LA length (cm)	4.60 (3.30, 5.40)	4.50 (3.00, 5.30)	5.00 (3.75, 5.82)	0.2
RA length (cm)	4.10 (2.60, 4.80)	4.00 (2.50, 4.70)	4.25 (3.25, 5.12)	0.2
T1 relaxation time (msec)	985 (951, 1,018)	986 (951, 1,018)	976 (953, 1,016)	0.7
T2 relaxation time (msec)	46.00 (45.00, 48.00)	46.50 (45.00, 48.00)	46.00 (44.00, 48.00)	0.5
Ascending aortic diameter (cm)	3.00 (2.60, 3.30)	3.00 (2.60, 3.20)	3.25 (2.88, 3.60)	0.002
**Functional imaging measures**				
LVEF	64 (59, 68)	64 (60, 68)	60 (55, 66)	**0**.**015**
RVEF	60 (56, 64)	60 (56, 64)	58 (50, 61)	**0**.**008**
LV output (L/min)	4.40 (3.70, 5.30)	4.40 (3.60, 5.10)	5.80 (4.27, 6.43)	**<0**.**001**
RV output (L/min)	3.90 (3.00, 4.80)	3.70 (2.80, 4.60)	4.85 (3.68, 6.10)	**<0**.**001**
**Stress imaging measures**				
Regadenoson stress	188 (83%)	156 (82%)	32 (89%)	0.3
Peak heart rate (BPM)	105 (93, 115)	105 (93, 116)	97 (92, 111)	**0**.**038**
Peak systolic blood pressure (mmHg)	124 (108, 140)	124 (110, 141)	116 (102, 137)	0.1
Myocardial perfusion reserve index	2.50 (2.10, 3.15)	2.50 (2.08, 3.10)	2.55 (2.10, 3.20)	0.9

LVEF, left ventricular ejection fraction; RVEF, right ventricular ejection fraction; LV, left ventricle, EDV, end diastolic volume; RV, right ventricle; LA, left atrium; RA, right atrium; Bold, *p* < 0.05.

Univariable linear regression of risk factors and MPRI did not demonstrate any significant associations in women. Conversely, in the male population, presence of hypertension (β: −0.61, *p* = 0.04), hyperlipidemia (β: −0.83, *p* = 0.005) or diabetes (β: −0.92, *p* = 0.03) were all associated with lower MPRI in univariable models, with diabetes (β: −0.80, *p* = 0.03) and hyperlipidemia (β: −0.76, *p* = 0.006) remaining associated with reduced MPRI in multivariable analysis (Table 2). Structural factors were also assessed, with univariable associations between increased aortic diameter (β: −0.44, *p* = 0.005) and longer T1 times (β: −0.0062, *p* = 0.01) in the male population; and lower LVEF (β: 0.017, *p* = 0.02), lower RVEF (β: 0.025, *p* = 0.006), higher LV EDV (β: −0.0052, *p* = 0.007), longer T1 times (β: −0.0032, *p* = 0.02) and higher LVMI (β: −0.022, *p* = 0.0004) associated with reduced MPRI in the female population. Final multivariable models revealed significant associations between reduced MPRI with higher ascending aortic diameter (β: −0.42, *p* = 0.005) and longer T1 times (β: −0.0056, *p* = 0.03) in the male population, and longer T1 times (β: −0.0037, *p* = 0.006) and higher LVMI (β: −0.022, *p* = 0.0003) in the female population (Table 3).

**TABLE 2:** Associations of traditional cardiovascular risk factors with myocardial perfusion reserve index (MPRI).

		Univariable model			Multivariable model		
		Beta	SE	*P*-value	Beta	SE	*P*-value
Male	Hypertension	-0.61	0.28	0.04	-	-	-
	Hyperlipidemia	-0.83	0.28	**<0**.**01**	-0.76	0.26	**0**.**01**
	Family history	0.01	0.39	0.98	-	-	-
	Diabetes	-0.92	0.39	**0**.**03**	-0.80	0.36	**0**.**03**
	Smoking	0.73	0.88	0.41	-	-	-
Female	Hypertension	-0.11	0.13	0.39	-	-	-
	Hyperlipidemia	-0.08	0.13	0.53	-	-	-
	Family history	0.10	0.14	0.47	-	-	-
	Diabetes	-0.06	0.28	0.83	-	-	-
	Smoking	-0.07	0.36	0.85	-	-	-

SE, standard error, Bold, *p* < 0.05.

**TABLE 3:** Associations of cardiac structure and function measures and the myocardial perfusion reserve index (MPRI).

		Univariable model			Multivariable model		
		Beta	SE	*P*-value	Beta	SE	*P*-value
Male	LVEF	0.00	0.02	0.95	-	-	-
	RVEF	0.00	0.02	0.79	-	-	-
	LV EDV	0.00	0.00	0.50	-	-	-
	RV EDV	0.00	0.00	0.29	-	-	-
	LA length	-0.13	0.06	0.05	-	-	-
	RA length	-0.10	0.07	0.16	-	-	-
	Ascending aortic diameter	-0.44	0.15	**<0**.**01**	-0.42	0.14	**<0**.**01**
	LV output	-0.06	0.09	0.53	-	-	-
	RV output	-0.07	0.07	0.27	-	-	-
	Relative wall thickness	0.20	0.28	0.49	-	-	-
	T1	-0.01	0.00	**0**.**03**	-0.01	0.00	**0**.**03**
	T2	-0.03	0.04	0.38	-	-	-
	LV mass index	-0.01	0.01	0.44	-	-	-
Female	LVEF	0.02	0.01	**0**.**02**	-	-	-
	RVEF	0.03	0.01	**0**.**01**	0.02	0.01	0.06
	LV EDV	-0.01	0.00	**<0**.**01**	-	-	-
	RV EDV	0.00	0.00	0.11	-	-	-
	LA length	0.00	0.02	0.88	-	-	-
	RA length	0.01	0.03	0.82	-	-	-
	Ascending aortic diameter	0.03	0.04	0.50	-	-	-
	LV output	-0.02	0.02	0.29	-	-	-
	RV output	0.00	0.01	0.85	-	-	-
	Relative wall thickness	-0.05	0.15	0.73	-	-	-
	T1	0.00	0.00	**0**.**02**	0.00	0.00	**<0**.**01**
	T2	0.01	0.02	0.66	0.03	0.02	0.14
	LV mass index	-0.02	0.01	**<0**.**001**	-0.02	0.01	**<0**.**001**

LVEF, left ventricular ejection fraction; RVEF, right ventricular ejection fraction; LV, left ventricle; EDV, end diastolic volume; RV, right ventricle; LA, left atrium; RA, right atrium. Bold values represent the *p* < 0.05.

Secondary analyses were performed in the aggregate population to assess the effects of choice of stress agent, and related physiological responses of peak heart rate and systolic blood pressure. Overall, regadenoson accounted for the majority of the stresses but the frequency was not significantly different between men and women. Peak systolic blood pressure was also not significantly different; however, we note that women had a higher peak heart rate response than men (Table 1). In the total population, in univariable analyses, stress agent choice (β: 0.98 for regadenoson vs. adenosine, *p* < 0.001) and peak systolic blood pressure (β: −0.0053, *p* = 0.049) were significantly associated with MPRI, with only stress agent being significant in multivariable analyses (β: 0.95, *p* < 0.001). Overall, the average MPRI with regadenoson was significantly higher than with adenosine [2.69 (2.20, 3.30) versus 1.80 (1.49, 2.10), *p* < 0.001]. Adjustment for stress agent in multivariable models with previously significant structural factors of T1 time and LVMI did not affect the significance of the factors (T1 time β: −0.0038, *p* < 0.001; LVMI β: −0.01, *p* = 0.01).

We also assessed the effects of structural factors on MPRI’s relation with LVMI in the aggregate population, specifically to understand whether “normalizing” the MPRI by LVMI to represent a relative flow per indexed myocardial tissue mass would reveal new associations between this normalized measure and other structural features. We assessed associations between MPRI, LVMI, and MPRI/LVMI with traditional risk factors and structural features using univariable models (Table 4). The associations of the individual factors (MPRI, LVMI) compared to MPRI normalized by LVMI were similar, i.e., the normalization of MPRI by LVMI did not draw out novel significant associations apart from previously significant associations from the individual factors (Table 4).

**TABLE 4:** Univariable associations of traditional risk factors and measures of cardiac structure and function with myocardial perfusion reserve index (MPRI), left ventricular mass index, and myocardial perfusion reserve index normalized by left ventricular mass index.

	MPRI			LV mass index			MPRI normalized by LV mass index		
	Beta	SE	*P*-value	Beta	SE	*P*-value	Beta	SE	*P*-value
Age	0.00	0.00	0.52	-0.06	0.06	0.34	0.00	0.00	0.35
Male sex	0.04	0.16	0.79	14.86	2.09	**<0**.**001**	-0.02	0.01	**<0**.**01**
Hypertension	-0.18	0.11	0.12	2.20	1.68	0.19	-0.01	0.00	0.12
Diabetes	-0.32	0.23	0.17	8.65	3.35	**0**.**01**	-0.01	0.01	0.23
Cholesterol	-0.18	0.11	0.12	-0.26	1.69	0.88	0.00	0.00	0.79
Family history	0.09	0.13	0.51	0.62	1.94	0.75	0.00	0.00	0.56
Smoking	0.05	0.33	0.89	-0.83	4.89	0.87	0.00	0.01	0.92
LVEF	0.01	0.01	**0**.**05**	-0.44	0.09	**<0**.**001**	0.00	0.00	**<0**.**01**
RVEF	0.02	0.01	0.05	-0.47	0.12	**<0**.**001**	0.00	0.00	**<0**.**01**
LV EDV	0.00	0.00	0.09	0.18	0.02	**<0**.**001**	0.00	0.00	**<0**.**001**
RV EDV	0.00	0.00	0.63	0.19	0.02	**<0**.**001**	0.00	0.00	**<0**.**001**
LA length	-0.01	0.02	0.66	0.03	0.30	0.92	0.00	0.00	0.81
RA length	-0.01	0.03	0.76	-0.35	0.41	0.40	0.00	0.00	0.60
Ascending aortic diameter	0.00	0.04	0.94	-0.10	0.53	0.85	0.00	0.00	0.80
LV output	-0.02	0.02	0.26	0.46	0.27	0.09	0.00	0.00	0.06
RV output	0.00	0.01	0.76	0.12	0.12	0.30	0.00	0.00	0.37
Relative wall thickness	0.01	0.13	0.96	3.74	1.93	0.05	0.00	0.00	0.38
T1	0.00	0.00	**<0**.**01**	-0.01	0.02	0.54	0.00	0.00	0.16
T2	0.00	0.02	0.97	0.31	0.27	0.24	0.00	0.00	0.55

LVEF, left ventricular ejection fraction; RVEF, right ventricular ejection fraction; LV, left ventricle; EDV, end diastolic volume; RV, right ventricle; LA, left atrium; RA, right atrium. Bold values represent the *p* < 0.05.

## Discussion

In this study, we assessed sex-differences in the relationships between traditional risk factors and features of cardiovascular structure and function with MPRI as a measure of CMD in patients without visible perfusion defects representing obstructive coronary disease. The main findings of our study were 3-fold. First, while there was no average difference between MPRI in the male and female groups, comparisons between MPRI’s associations in men and women revealed notable differences. Specifically, associations with hyperlipidemia, diabetes, T1 times and ascending aortic diameter were seen in men, whereas women had no associations with traditional risk factors, but had associations with T1 times and LVMI. Secondly, use of regadenoson was associated with higher MPRI than adenosine but did not appear to affect other associations. Finally, normalization by structural features did not reveal novel associations.

There is a robust historical literature of differences in cardiovascular disease between men and women, which is in part mediated by the complex relations between genetic determinants, risk exposures and the relative impact of risk factors over time, and different cardiovascular aging trajectories (13). Within the coronary microvasculature, less is known. Most literature cites higher prevalence of systemic and macrovascular coronary vasomotor disorders, as well as differences neurological mediation of coronary function in women, which theoretically may lead toward CMD and subsequent coronary rarefaction contributing to HFpEF (14–17). Given the high diversity of phenotypes contained within CMD, these explanations seem to provide an incomplete accounting for differences between women and men. The lack of associations between MPRI and traditional risk factors in the female population seems to support these findings, in that traditional risk factors may not accurately reflect risk of vasomotor disorders and neurological mediation (18). This is juxtaposed against the unique associations seen in men, specifically hyperlipidemia and diabetes, which is more in line with “traditional” atherosclerotic cardiovascular disease, as is aortic dilation as a presentation of vascular disease which may integrate cardiovascular multiple risk factors.

While PET provides quantitative analysis of absolute blood flow, not yet widely available with CMR, and is more widely used than CMR for identification of CMD, CMR has the benefit of providing comprehensive myocardial information including structure, function, and composition. The findings of increased T1 times and LVMI being associated with reduced MPRI in the overall and female populations, as well as T1 times in the male population is useful for understanding potential associated pathophysiology of CMD. Increased T1 time may be representative of increased myocardial fibrosis, and increased LVMI pushes toward hypertrophic phenotypes, either eccentric or concentric, though we note with some surprise that relative wall thickness was not associated with MPRI. These findings suggest subclinical myocardial injury with remodeling and fibrosis accompanying reduced MPRI. As opposed to risk factors, the potential direction of causality between exposures and outcomes is uncertain. CMD may be causing the remodeling and fibrosis (e.g., as progression toward HFpEF), or the converse may be true with the fibrotic replacement and pathological remodeling resulting in damage to the coronary microvasculature.

We also noted that the stress agent itself appear to have a distinct effect, with regadenoson being associated with higher MPRI values than adenosine. Most prior literature suggests in the context of coronary perfusion testing, regadenoson and adenosine induce similar degrees of hyperemia, (19–21) with two exceptions which showed regadenoson inducing higher flows than adenosine (22, 23). As an observational cohort, we recognize that there may be some degree of confounding by indication, as regadenoson is typically considered safer in patients with history of reactive airway disease; however, these findings suggest some degree of caution for direct comparison of MPRI between the two different stress agents.

We attempted to further understand whether normalizing MPRI by LVMI had unique value or second order relations, as it may be biologically reasonable to associate higher degrees of perfusion with higher myocardial mass. These analyses were unrevealing and did not show any new associations with structure/function or risk factors compared to MPRI or LVMI alone. This may be explained by the fact that MPRI is calculated as a ratio of ratios: stress (myocardial signal/blood pool signal)/rest (myocardial signal/blood pool signal). Given that cardiac structural parameters should remain constant at stress and rest, one would expect them to have no net effect on MPRI.

Our study has several limitations. First, as a single-center clinically referred cohort, there may be some degree of selection bias within our population. Our included population was predominantly female, and the reduced male sample size may have limited our power to identify more male-specific findings related to MPRI, which may be discovered by analysis of larger male populations. Additionally, while exclusion of cases with visible perfusion defects helps eliminate patients with obstructive disease, we are unable to differentiate between the patients who have no coronary atherosclerosis versus those with non-obstructive disease, which may represent different CMD phenotypes. While MPRI is well-validated for CMD, it is a semi-quantitative measure of perfusion due to signal saturation within the blood pool. Fully quantitative measures of perfusion may yield different results, though guideline-based consensus on optimal approach to quantitative CMR perfusion has not yet been reached at this time. Finally, while invasive measurement of coronary microcirculation may provide a gold-standard measurement of microvascular dysfunction, this is rarely performed in a clinical context within our institution and therefore we lack direct invasive confirmation of microvascular dysfunction within our patients. Recent advances in invasive methods have been developed including alternative stress agent use and continuous intracoronary thermodilution-derived methods, which may accelerate acquisition and may improve upon non-invasive diagnostic evaluations (24–28). Confirmation of our results using gold-standard invasive measures should be considered.

In conclusion, MPRI as a marker of CMD is not associated with traditional cardiovascular risk factors in women but associated with hyperlipidemia and diabetes in men. MPRI is associated with T1 times and LVMI in women, and T1 times and aortic diameter in men. These findings suggest different underlying pathophysiology of CMD in men versus women, with reductions in MPRI in male patients fitting a more “traditional” atherosclerotic profile. Further research to understand the underlying pathophysiology between sex differences in CMD is necessary.

## Data availability statement

The original contributions presented in this study are included in this article/supplementary material, further inquiries can be directed to the corresponding author.

## Ethics statement

The studies involving human participants were reviewed and approved by Cedars Sinai Institutional Review Board. Written informed consent for participation was not required for this study in accordance with the national legislation and the institutional requirements.

## Author contributions

AK, JW, CM, DB, and SC contributed to conception and design of the work. AK, JE, and DB contributed to the acquisition of the data. AK, DO, JE, and SC performed the analysis and interpretation of the data. AK wrote the initial draft. All authors provided comprehensive edits, approved the publication of the content, and agreed to accountability for the work and approved the submitted version.

## References

[1] Camici P Crea F. Coronary microvascular dysfunction. *N Eng J Med.* (2007) 356:830–40. 10.1056/NEJMra061889 17314342

[2] Mileva N Nagumo S Mizukami T Sonck J Berry C Gallinoro E Prevalence of coronary microvascular disease and coronary vasospasm in patients with nonobstructive coronary artery disease: systematic review and meta-analysis. *J Am Heart Assoc.* (2022) 11:e023207. 10.1161/JAHA.121.023207 35301851 PMC9075440

[3] Padro T Manfrini O Bugiardini R Canty J Cenko E De Luca G ESC working group on coronary pathophysiology and microcirculation position paper on ‘coronary microvascular dysfunction in cardiovascular disease’. *Cardiovasc Res.* (2020) 116:741–55. 10.1093/cvr/cvaa003 32034397 PMC7825482

[4] Sara J Widmer R Matsuzawa Y Lennon R Lerman L Lerman A. Prevalence of coronary microvascular dysfunction among patients with chest pain and nonobstructive coronary artery disease. *Cardiovasc Inter.* (2015) 8:1445–53. 10.1016/j.jcin.2015.06.017 26404197

[5] Murthy V Naya M Taqueti V Foster C Gaber M Hainer J Effects of sex on coronary microvascular dysfunction and cardiac outcomes. *Circulation.* (2014) 129:2518–27. 10.1161/CIRCULATIONAHA.113.008507 24787469 PMC4076200

[6] Taqueti V Solomon S Shah A Desai A Groarke J Osborne M Coronary microvascular dysfunction and future risk of heart failure with preserved ejection fraction. *Eur Heart J.* (2018) 39:840–9. 10.1093/eurheartj/ehx721 29293969 PMC5939665

[7] Giamouzis G Schelbert E Butler J. Growing evidence linking microvascular dysfunction with heart failure with preserved ejection fraction. *Am Heart Assoc.* (2016) 5:e003259. 10.1161/JAHA.116.003259 26908416 PMC4802456

[8] Taqueti V Carli M. Coronary microvascular disease pathogenic mechanisms and therapeutic options. *J Am Coll Cardiol.* (2018) 72:2625–41. 10.1016/j.jacc.2018.09.042 30466521 PMC6296779

[9] Bakir M Wei J Nelson M Mehta P Haftbaradaran A Jones E Cardiac magnetic resonance imaging for myocardial perfusion and diastolic function-reference control values for women. *Cardiovasc Diagn Ther.* (2016) 6:78–86. 26885495 10.3978/j.issn.2223-3652.2015.09.03 PMC4731584

[10] Shufelt C Thomson L Goykhman P Agarwal M Mehta P Sedlak T Cardiac magnetic resonance imaging myocardial perfusion reserve index assessment in women with microvascular coronary dysfunction and reference controls. *Cardiovasc Diagn Ther.* (2013) 3:153–60. 24282764 10.3978/j.issn.2223-3652.2013.08.02 PMC3839208

[11] Thomson L Wei J Agarwal M Haft-Baradaran A Shufelt C Mehta P Cardiac magnetic resonance myocardial perfusion reserve index is reduced in women with coronary microvascular dysfunction. *Circulation Cardiovasc Imaging.* (2015) 8:e002481. 10.1161/CIRCIMAGING.114.002481 25801710 PMC4375783

[12] Wöhrle J Nusser T Merkle N Kestler H Grebe O Marx N Myocardial perfusion reserve in cardiovascular magnetic resonance: correlation to coronary microvascular dysfunction. *J Cardiovas Mag Res.* (2006) 8:781–7. 10.1080/10976640600737649 17060099

[13] Ji H Kwan A Chen M Ouyang D Ebinger J Bell S Sex differences in myocardial and vascular aging. *Circ Res.* (2022) 130:566–77. 10.1161/CIRCRESAHA.121.319902 35175845 PMC8863105

[14] Paul T Sivanesan K Schulman-Marcus J. Sex differences in nonobstructive coronary artery disease: recent insights and substantial knowledge gaps. *Trends Cardiovas Med.* (2017) 27:173–9. 10.1016/j.tcm.2016.08.002 27617797

[15] Groepenhoff F Bots S Kessler E Sickinghe A Eikendal A Leiner T Sex-specific aspects in the pathophysiology and imaging of coronary macro- and microvascular disease. *J Cardiovasc Trans Res.* (2020) 13:39–46. 10.1007/s12265-019-09906-0 31471830 PMC7010630

[16] Waheed N Elias-Smale S Malas W Maas A Sedlak T Tremmel J Sex differences in non-obstructive coronary artery disease. *Cardiovasc Res.* (2020) 116:829–40. 10.1093/cvr/cvaa001 31958135

[17] Vaccarino V Badimon L Corti R de Wit C Dorobantu M Hall A Ischaemic heart disease in women: are there sex differences in pathophysiology and risk factors?: position paper from the working group on coronary pathophysiology and microcirculation of the European society of cardiology. *Cardiovasc Res.* (2010) 90:9–17. 10.1093/cvr/cvq394 21159671 PMC3058737

[18] Reynolds H Bairey Merz C Berry C Samuel R Saw J Smilowitz N Coronary arterial function and disease in women with no obstructive coronary arteries. *Circulat Res.* (2022) 130:529–51. 10.1161/CIRCRESAHA.121.319892 35175840 PMC8911308

[19] Kero T Saraste A Lagerqvist B Sörensen J Pikkarainen E Lubberink M Quantitative myocardial perfusion response to adenosine and regadenoson in patients with suspected coronary artery disease. *J Nuclear Cardiol.* (2022) 29:24–36. 10.1007/s12350-021-02731-6 34386859 PMC8873130

[20] Iskandrian A Bateman T Belardinelli L Blackburn B Cerqueira M Hendel R Adenosine versus regadenoson comparative evaluation in myocardial perfusion imaging: results of the ADVANCE phase 3 multicenter international trial. *J Nuclear Cardiol.* (2007) 14:645–58. 10.1016/j.nuclcard.2007.06.114 17826318

[21] Stolker J Lim M Shavelle D Morris D Angiolillo D Guzman L Pooled comparison of regadenoson versus adenosine for measuring fractional flow reserve and coronary flow in the catheterization laboratory. *Cardiovasc Revasc Med.* (2015) 16:266–71. 10.1016/j.carrev.2015.05.011 26242981

[22] Vasu S Bandettini W Hsu L Kellman P Leung S Mancini C Regadenoson and adenosine are equivalent vasodilators and are superior than dipyridamole- a study of first pass quantitative perfusion cardiovascular magnetic resonance. *J Cardiovas Magn Reson.* (2013) 15:85. 10.1186/1532-429X-15-85 24063278 PMC3851492

[23] Wei J Jalnapurkar S Dela Cruz S Cook-Wiens G Motwani M Zhang X Adenosine vs regadenoson pharmacologic stress differs in women with suspected coronary microvascular dysfunction: a report from the Women’s ischemia syndrome evaluation-coronary vascular dysfunction (WISE-CVD) study. *Cardiovasc Disord Med.* (2019) 1:1–7. 10.31487/j.CDM.2019.01.01 PMC9997839 36913201

[24] Kodeboina M Nagumo S Munhoz D Sonck J Mileva N Gallinoro E Simplified assessment of the index of microvascular resistance. *J Interv Cardiol.* (2021) 2021:9971874. 10.1155/2021/9971874 34149324 PMC8189791

[25] Gallinoro E Paolisso P Candreva A Bermpeis K Fabbricatore D Esposito G Microvascular dysfunction in patients with type ii diabetes mellitus: invasive assessment of absolute coronary blood flow and microvascular resistance reserve. *Front Cardiovasc Med.* (2021) 8:765071. 10.3389/fcvm.2021.765071 34738020 PMC8562107

[26] Candreva A Gallinoro E Fernandez Peregrina E Sonck J Keulards D Van’t Veer M Automation of intracoronary continuous thermodilution for absolute coronary flow and microvascular resistance measurements. *Catheter Cardiovasc Interv.* (2022) 100:199–206. 10.1002/ccd.30244 35723684

[27] Vandeloo B Andreini D Brouwers S Mizukami T Monizzi G Lochy S Diagnostic performance of exercise stress tests for detection of epicardial and microvascular coronary artery disease: the UZ Clear study. *Eur Interv.* (2022). 10.4244/EIJ-D-22-00270 [Epub ahead of print]. 36147027 PMC9909457

[28] Gallinoro E Paolisso P Vanderheyden M Esposito G Bertolone DT Mileva N Assessment of absolute coronary flow and microvascular resistance reserve in patients with severe aortic stenosis. *Eur Heart J.* 43:ehac544.2023. 10.1093/eurheartj/ehac544.2023 35977812

